# Retrospective Motion Artifact Reduction by Spatial Scaling of Liver Diffusion-Weighted Images

**DOI:** 10.3390/tomography9050146

**Published:** 2023-10-06

**Authors:** Johannes Raspe, Felix N. Harder, Selina Rupp, Sean McTavish, Johannes M. Peeters, Kilian Weiss, Marcus R. Makowski, Rickmer F. Braren, Dimitrios C. Karampinos, Anh T. Van

**Affiliations:** 1 School of Medicine and Health, Technical University of Munich, 81675 Munich, Germanydimitrios.karampinos@tum.de (D.C.K.); anh.van@tum.de (A.T.V.); 2 School of Natural Sciences, Technical University of Munich, 85748 Garching, Germany; 3 Philips Healthcare, 5684 PC Best, The Netherlands; 4 Philips GmbH Market DACH, 22335 Hamburg, Germany

**Keywords:** liver, magnetic resonance imaging (MRI), diffusion weighted imaging (DWI), cardiac motion, artifact, intravoxel dephasing induced signal loss, apparent diffusion coefficient (ADC) bias

## Abstract

Cardiac motion causes unpredictable signal loss in respiratory-triggered diffusion-weighted magnetic resonance imaging (DWI) of the liver, especially inside the left lobe. The left liver lobe may thus be frequently neglected in the clinical evaluation of liver DWI. In this work, a data-driven algorithm that relies on the statistics of the signal in the left liver lobe to mitigate the motion-induced signal loss is presented. The proposed data-driven algorithm utilizes the exclusion of severely corrupted images with subsequent spatially dependent image scaling based on a signal-loss model to correctly combine the multi-average diffusion-weighted images. The signal in the left liver lobe is restored and the liver signal is more homogeneous after applying the proposed algorithm. Furthermore, overestimation of the apparent diffusion coefficient (ADC) in the left liver lobe is reduced. The proposed algorithm can therefore contribute to reduce the motion-induced bias in DWI of the liver and help to increase the diagnostic value of DWI in the left liver lobe.

## 1. Introduction

Diffusion-weighted (DW) magnetic resonance imaging (MRI) [1,2] depicts tissue microstructure through probing the molecular diffusion process [3]. It has crucial, established applications in brain imaging, both in neuroscience and neurological imaging [4,5,6,7]. Translation of diffusion-weighted imaging (DWI) to the body is desirable and has been performed in multiple organs including the liver [8,9,10,11]. In the liver, DWI has been shown to be useful for the detection and characterization of focal hepatic lesions [12,13,14], for the assessment of treatment response [9,15], and for the evaluation of diffuse liver disease [16,17].

DWI is influenced by multiple sources of systematic errors, including eddy current effects [18] and gradient non-linearities [19,20] as well as other higher-order effects, such as the spatial dependence of b-values [21]. In liver applications, DWI is prone to more severe sources of error, including complex motion effects [9,22,23], worse B0 and B1 inhomogeneity [24], difficult fat suppression [25], and shorter tissue T2 relaxation [26,27]. Types of motion that affect the liver include cardiac [9,23,28,29], respiratory [9,28], and gross body motion [9]. In the presence of diffusion encoding gradients, motion leads to spin phase accumulation. At the voxel level, this motion-induced spin phase accumulation results in intra-voxel dephasing and/or a voxel phase. When intra-voxel dephasing occurs due to non-uniform motion at the voxel scale, the affected diffusion-weighted images exhibit non-diffusion-induced signal loss, confounding the estimated diffusion parameters [23,30,31]. When motion leads to an additional voxel phase, the effect is commonly ignored when a single-shot acquisition is employed since only the magnitude images are typically needed.

Many studies have investigated and compared different methods for minimizing the effect of motion, especially respiratory motion, in liver DWI [28,30,32,33,34]. Breath-holding and respiratory triggering are the most widely used methods in liver DWI for reducing respiratory motion effects [28,33,35]. Breath-holding performance can vary depending on the cooperation and the fitness of the subject and might not be suitable for all patient scans. The performance of respiratory triggering depends on the stability of the breathing pattern and therefore warrants residual motion effects. With respect to cardiac motion, although cardiac gating can mitigate cardiac motion effects, including in the left liver lobe [32,36], the inefficiency of a cardiac-gated scan prevents it from being widely applied [9]. In recent years, motion-compensated diffusion encoding (MCDE) waveforms have emerged as an additional method for minimizing motion in liver DWI [20,37,38,39]. MCDE can reduce the effect of motion regardless of its sources. However, MCDE waveforms result in a prolonged echo time (TE) that might prevent their application, especially in patients with a short liver T2. The prolonged TE and the accompanied flow-compensation properties of MCDE waveforms also brighten up the vessel signal in liver DWI, confounding estimated apparent diffusion coefficient (ADC) maps and compromising diagnostic value [20,39]. Reduction of the vessel signal requires a reduction in the motion compensation capability [39].

Post-processing techniques have also been proposed to mitigate motion-induced signal loss in liver DWI. Chang et al. [40] implemented an algorithm rejecting outlier data. However, eliminating data might worsen the noise performance of the method if the rejected data are above the noise floor. By employing power-of-four averaging instead of the standard sum of magnitudes (equivalent to power-of-one averaging), Liau et al. [29] showed that the cardiac-motion-induced signal loss and the discrepancy of ADC between the left and right liver lobes are reduced. However, increasing the power of averaging also leads to an increase in noise in the averaged image. Furthermore, the optimum power changes with individual subjects and b-values, and there is no systematic method for choosing the proper power. Weighted averaging was also proposed to reduce signal loss in the left liver lobe in liver DWI [41]. The weights were computed per diffusion encoding direction by dividing the square of individual averages by the sum of squares of all averages. Algorithms similar to all of the aforementioned ones were compared by Führes et al. [42] when using MCDE waveforms. A weighted averaging approach using low-pass-filtered weight maps was shown to increase image quality most in terms of minimizing pulsation artifacts and increasing the lesion contrast-to-noise ratio while maintaining data consistency.

More recently, deep learning approaches have also been introduced to perform weighted averaging of liver DWI. In Gadjimuradov et al. [43], a network was trained to compute the weights for a weighted averaging. The same authors developed another method that uses a sliding window for adaptive weighting based on small patches of multiple repetitions [44]. They also used a trained classifier to predict whether an average contains significant signal dropout to robustly estimate a good reference value to compare to.

The purpose of the present work was to develop a model and algorithm to tackle the problem of motion-induced signal loss in liver DWI. Therefore, the present work proposes a novel algorithm that reduces the motion-induced signal loss in respiratory-triggered liver DWI. It combines the rejection of heavily corrupted data and spatial scaling of the average diffusion-weighted images. It therefore can potentially increase the diagnostic quality of liver diffusion data. Data rejection was performed at the repetition and voxel levels and was incorporated before the averaging. The proposed algorithm was tested on in vivo DWI data from patients receiving an abdominal MRI examination.

## 2. Materials and Methods

### 2.1. Implicit Weighted Averaging

To improve the signal-to-noise ratio (SNR) and mitigate motion artifacts, diffusion imaging often acquires multiple repetitions for each b-value and diffusion encoding direction. Let $\left\{I_{z,i}\left(x,y\right)|i=1,\;\ldots ,\;N\right\}$ be the magnitude of the DW image at the in-plane spatial location (x,y) from the *i*-th repetition of slice *z* for each b-value. The average DW image is conventionally computed as the sum of the magnitudes of all averages as

(1) $$\mu_{z,\;\mathrm{standard}}\left(x,y\right)=\frac{\sum_{i=1}^{N}I_{z,i}\left(x,y\right)}{N},$$

though other conventions, such as a combination of geometric and arithmetic averages, exist [42].

**Figure 1:**
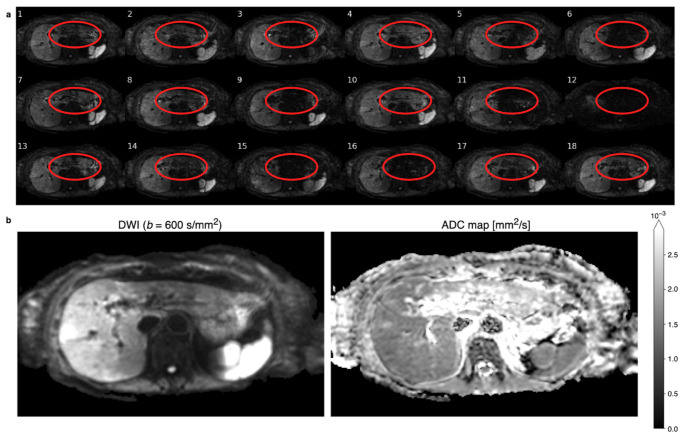
(**a**) Six repetitions of respiratory-triggered diffusion-weighted images (DWIs) with a b-value of $b=600\;{s/\mathrm{mm}}^{2}$ for three different diffusion directions that show different patterns of motion-induced signal-loss artifacts. The location of the left liver lobe is circled in red for each repetition. Some repetitions, like number 12, show global signal loss, while more often, local signal loss in the left liver lobe is observable, e.g., repetition 15. The local signal loss is incoherent and unpredictable between repetitions. (**b**) A sum of magnitudes of the 18 repetitions yields the diffusion-weighted (DW) image on the left that displays an inhomogeneous liver with evidently higher values in the right liver lobe. Apparent diffusion coefficient (ADC) mapping calculated using the motion-affected DWIs results in a inhomogeneous appearance of ADC in the liver.

An example of 18 repetitions of liver DWI at a b-value of $b=600\;{s/\mathrm{mm}}^{2}$ is depicted in Figure 1a, wherein the loss of signal in some repetitions—especially in the left liver lobe—is apparent. The sum of magnitudes of these images as in Equation (1) leads to an average diffusion-weighted image with an inhomogeneous liver, which is depicted in Figure 1b on the left. An ADC map calculated from motion-corrupted images typically manifests overestimation in the left liver, as shown on the right in Figure 1b.

The motion-induced signal loss as observable in multiple repetitions in Figure 1a can be viewed as repetition- and spatially dependent signal weighting. Thus, the magnitude of the obtained image can be expressed as

(2) $$I_{z,i}\left(x,y\right)=w_{z,i}\left(x,y\right){\hat{I}}_{z}\left(x,y\right)+n_{z}\left(x,y\right),$$

where $w_{z,i}\left(x,y\right)$ denotes the weighting factor, ${\hat{I}}_{z}\left(x,y\right)$ is the image magnitude without motion-induced signal loss that is independent of repetitions, and $n_{z}\left(x,y\right)$ is a noise term. For simplicity, Equation (2) only considers the motion-induced signal fluctuation across repetitions. Other changes across repetitions—induced by misregistration, phase variation, and distortion—are ignored. Substituting Equation (2) into Equation (1) yields

(3) $$\mu_{z,\;\mathrm{standard}}\left(x,y\right)=\frac{\sum_{i=1}^{N}w_{z,i}\left(x,y\right){\hat{I}}_{z}\left(x,y\right)+n_{z}\left(x,y\right)}{N}.$$

Motion-induced signal loss in DWI results in $\sum_{i=1}^{N}w_{z,i}\left(x,y\right)<N$ and therefore in incorrectly smaller values of the estimated average DW image. Ignoring noise, if the signal weighting factor $w_{z,i}\left(x,y\right)$ is known, the true image magnitude can be estimated as

(4) $${\hat{I}}_{z}\left(x,y\right)\approx \frac{\sum_{i=1}^{N}I_{z,i}\left(x,y\right)}{W_{z}\left(x,y\right)},$$

where

(5) $$W_{z}\left(x,y\right)=\sum_{i=1}^{N}w_{z,i}\left(x,y\right).$$

### 2.2. Spatial Scaling for the Reduction of Motion-Induced Signal Loss

**Figure 2:**
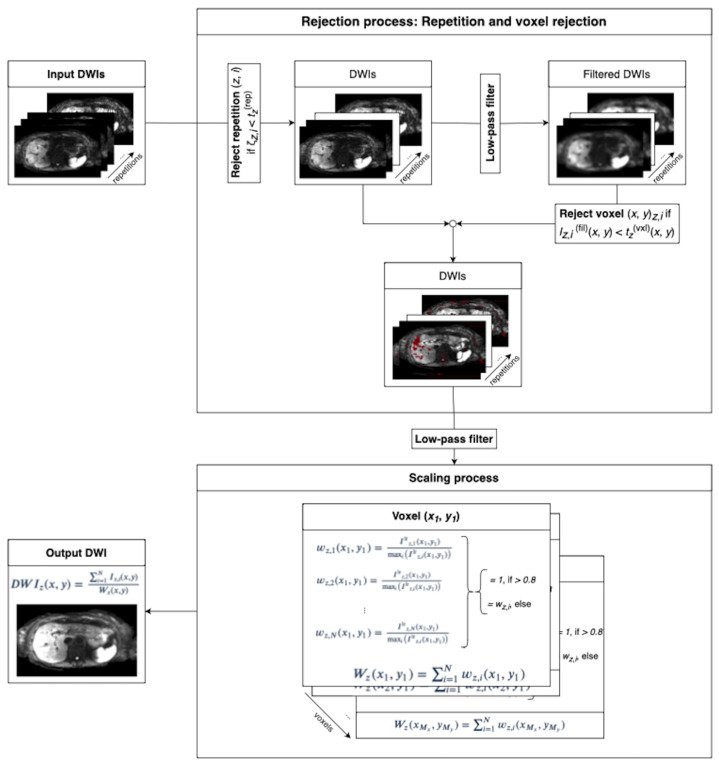
The two-step algorithm takes repetitions of one slice and one b-value as input. DW images in different directions are treated as additional repetitions. The sum of intensities of all voxels in each repetition is compared to a threshold ${t_{z}}^{\left(\mathrm{rep}\right)}$ for repetition rejection. Subsequent low-pass filtering blurs the images to consider that motion artifacts tend to affect patches of the image in the voxel rejection step. Spatially dependent thresholding on the filtered images determines whether a voxel is rejected. The spatially dependent weights $w_{z,i}\left(x,y\right)$ are computed on low-pass-filtered data by dividing the intensity of each repetition by the maximum intensity projection across all repetitions. Weights that exceed a value of 0.8 are set to 1. The resulting DW image is found by scaling the sum across repetitions of the magnitude of non-rejected voxels with the spatially dependent scaling factor $W_{z}\left(x,y\right)$ according to Equation (4).

The proposed algorithm is an extension of a previously presented conference abstract [45]. It involves two primary processes: first, rejection of repetitions and voxel outliers, and second, spatially dependent magnitude-based scaling of the average DW image. The overall workflow of the algorithm is depicted in Figure 2 and is applied to all repetitions and slices of a single b-value as well as for all b-values of interest. In addition, the isotropic diffusivity assumption of diffusion in the liver parenchyma [46] enables the treatment of diffusion encoding directions as additional repetitions, thereby providing better statistics for the algorithm. Thus, the total number of repetitions *N* in this work is a product of the number of repetitions per diffusion direction and the number of diffusion encoding directions.

#### 2.2.1. Rejection Process

The rejection process is divided into two steps: repetition rejection and voxel rejection. Complete repetitions are only rejected when they contain little to no valuable information that adds to the resulting DW image. An example of a repetition that should be rejected is repetition number 12 in Figure 1a. Whether the *i*-th repetition of slice *z*, $I_{z,i}$, gets rejected is determined by thresholding. $I_{z,i}$ gets rejected if the sum of intensities $\zeta_{z,i}=\sum_{\left(x,y\right)}I_{z,i}\left(x,y\right)$ of all of its voxels is below the threshold

(6) $${t_{z}}^{\left(\mathrm{rep}\right)}=\underset{i}{\mathrm{med}}\left(\zeta_{z}\right)-\lambda^{\left(\mathrm{rep}\right)}\underset{i}{\mathrm{mad}}\left(\zeta_{z}\right),$$

where $\zeta_{z}={\left\{\zeta_{z,i}\right\}}_{i}$ is the set of sums of intensities of all repetitions of slice *z*, $\lambda^{\left(\mathrm{rep}\right)}$ is a thresholding parameter, med() denotes the median [47], and mad() denotes the median absolute deviation (MAD) [48,49]. The MAD represents a more robust measure of data dispersion as compared to the standard deviation in the presence of outliers [49]. The median absolute deviation is defined by

(7) $$\underset{i}{\mathrm{mad}}\left(\zeta_{z}\right)=\underset{i}{\mathrm{med}}\left(\left\{\left|\zeta_{z,i}-\underset{j}{\mathrm{med}}\left(\left\{\zeta_{z}\right\}\right)\right|\right\}\right),$$

where *i* and *j* are repetition indices and *z* is the slice index. The variable $\lambda^{\left(\mathrm{rep}\right)}$ in Equation (6) decides the distance of the threshold from the median value ${\mathrm{med}}_{i}\left(\zeta_{z}\right)$. Since only repetitions that are totally corrupted and have almost no signal are targeted, $\lambda^{\left(\mathrm{rep}\right)}$ should be positive: $\lambda^{\left(\mathrm{rep}\right)}>0$ implies that at most half of the repetitions from one slice are rejected. In the absence of motion-corrupted repetitions, all repetitions of the slice in consideration are not affected by motion-induced signal loss and their magnitude fluctuation should be caused primarily by noise. By choosing a threshold that is multiple MADs below the median, the possibility of rejecting slices unaffected by signal loss is low. When all repetitions of the slice in consideration are heavily corrupted by motion-induced signal loss, the aforementioned repetition rejection step cannot provide better performance than standard averaging, meaning the slice in consideration is corrupted in the final average DW image. In our implementation, $\lambda^{\left(\mathrm{rep}\right)}$ is fixed to the empirical value of 5, which means the threshold is five times the MAD below the median. This value marks a conservative compromise between rejection of slices with valuable information and not rejecting slices with total signal loss. “Conservative” in this situation refers to the choice of a threshold parameter rather greater than smaller in order to avoid over-rejection. Rejecting too little data can in part be compensated by the scaling described in Section 2.2.2, while over-rejection will lead to irreversible loss of data.

The second step focuses on rejecting outlier voxels that experience severe signal loss. The voxel rejection step targets localized motion-induced signal loss mainly caused by cardiac motion. Examples of the local signal loss can be seen in Figure 1a, e.g., repetitions number 6 and 15. Despite being localized, the motion-induced signal loss should affect a neighborhood of voxels instead of individual voxels. Thus, a low-pass filter is applied to the image before the voxel rejection step to utilize the fact that motion artifacts tend to affect patches of an image. A voxel ${\left(x,y\right)}_{z,i}$ at slice *z* of the low-pass-filtered repetition *i* gets rejected if its magnitude is below the threshold

(8) $${t_{z}}^{\left(\mathrm{vxl}\right)}(x,y)=\underset{i}{\mathrm{med}}\left({I^{\mathrm{lr}}}_{z}\left(x,y\right)\right)-\lambda^{\left(\mathrm{vxl}\right)}\underset{i}{\mathrm{mad}}({I^{\mathrm{lr}}}_{z}\left(x,y\right)),$$

where ${I^{\mathrm{lr}}}_{z}$ is the set of all low-resolution repetitions of slice *z* and $\lambda^{\left(\mathrm{vxl}\right)}$ is an adjustable thresholding parameter. It is worth noting that the threshold ${t_{z}}^{\left(\mathrm{vxl}\right)}\left(x,y\right)$ is spatially dependent. Similar to $\lambda^{\left(\mathrm{rep}\right)}$, $\lambda^{\left(\mathrm{vxl}\right)}$ is fixed to an empirical value of 3 in the present algorithm.

#### 2.2.2. Spatial Scaling of Average Diffusion-Weighted Images

The spatial scaling factor $w_{z,i}\left(x,y\right)$ in Equation (2) represents the motion-induced signal change map of each repetition as compared to the ground truth image. Since motion during diffusion encoding leads predominantly to signal loss, the maximum intensity projection (MIP) of all repetitions can approximate the ground truth image. In this case, for each slice *z*, repetition *i*, and in-plane location (x,y), the weighting factor $w_{z,i}\left(x,y\right)$ can be computed as

(9) $$w_{z,i}\left(x,y\right)=\frac{{I^{\mathrm{lr}}}_{z,i}\left(x,y\right)}{\max_{i}\left({I^{\mathrm{lr}}}_{z,i}\left(x,y\right)\right)},$$

where ${I^{\mathrm{lr}}}_{z,i}$ is the *i*-th repetition of slice *z* with a low-pass filter applied. This filtering step before the weight computation assures better noise performance in the scaled image. The scaling factors are computed for non-rejected voxels only and are set to 0 for rejected voxels. To reduce the underestimation of the scaling factors and subsequent overestimation of the average image magnitude due to noise, scaling factors that exceed a value of 0.8 are set to 1. The final scaled average DW image of slice *z* is

(10) $$DWI_{z}\left(x,y\right)=\frac{\sum_{i=1}^{N}I_{z,i}\left(x,y\right)}{\sum_{i=1}^{N}w_{z,i}\left(x,y\right)}=\frac{\sum_{i=1}^{N}I_{z,i}\left(x,y\right)}{W_{z}\left(x,y\right)}.$$

### 2.3. Data Acquisition

A total of 72 patients receiving an abdominal MRI examination were scanned on the 3 T Ingenia Elition X whole-body scanner by Philips Healthcare (Best, The Netherlands) using a 16-channel torso coil and the build-in-table 12-channel posterior coil. The local ethics committee approved the study. A spin-echo DW sequence with a single-shot echo-planar imaging (EPI) readout was employed with the following acquisition parameters: field of view FOV=(420×370) mm^2^, voxel size of (3×3×4) mm^3^; 60 slices with 0.4 mm gap; b-values of $b=\left(0,\;50,\;300,\;600\right)\;{s/\mathrm{mm}}^{2}$ with 2, 1, 2, and 6 repetitions, respectively; and 3 diffusion encoding directions for $b>0\;{s/\mathrm{mm}}^{2}$. All scans were respiratory triggered with a fixed trigger delay of 200 ms.

The data were analyzed retrospectively for the purpose of the present work. Therefore, this work relies on the analysis of routine clinical DW image data from patients in the institution receiving diagnostic abdominal MRI for a wide range of pathologies.

### 2.4. Image Reconstruction and Analysis

Reconstructions were performed offline using standard averaging (Equation (1)) and spatially scaled averaging (Equation (10)) with prior outlier rejection. A mono-exponential model was applied on the average DWIs at $b=\left(50,\;300,\;600\right)\;{s/\mathrm{mm}}^{2}$ to estimate ADC maps.

The offline reconstruction was performed using MATLAB by MathWorks (Natick, MA, USA) [50] and the ReconFrame framework by GyroTools (Zurich, Switzerland). The proposed algorithm was implemented in Python [51], including the external libraries NumPy [52], SciPy [53], Astropy [54,55], Matplotlib [56], and HDF5 for Python [57].

#### 2.4.1. ADC Comparison

For ADC comparison between standard averaging and the proposed method—composed of outlier rejection and spatially scaled averaging—circular regions of interest (ROIs) were placed in the nine Couinaud liver segments [58]. Care was taken to avoid including pathological regions and big vessels in the ROIs. Segments I, II, III, IVa, and IVb were assigned to the left liver lobe, and the remaining segments V, VI, VII, and VIII were assigned to the right liver lobe [39,58,59].

ADC evaluation was also performed in the paraspinal muscle region—where there is minimal motion-induced signal loss—to evaluate the bias of the proposed method. Since diffusion in the muscle is anisotropic, diffusion encoding directions were not considered as additional averages in the application of the spatially scaled averaging algorithm for the muscle region. Instead, each diffusion encoding direction was treated independently. Two ROIs in the left and right sides of the paraspinal muscle of each patient were drawn.

#### 2.4.2. Clinical Evaluation

A radiologist blindly evaluated and compared average DWIs at a b-value of $b=600\;{s/\mathrm{mm}}^{2}$ from standard averaging and the proposed method. Four non-diagnostic data sets and one that only partly covered the liver were excluded, leaving 67 data sets for evaluation. The comparison criteria were

1. Overall image quality: the gross appearance of the whole image volume;
2. Liver homogeneity: the contrast between the signal of the liver parenchyma of the left lobe versus the right lobe;
3. Perceived signal-to-noise ratio (SNR): the visual perception of the noise performance;
4. Quality of lesion detection: the possibility to distinguish healthy liver parenchyma from lesions.

The scores for each criterion ranged from ranged from 1 to 4, with 4 being the best score. The ADC images were only checked in the case of at least one lesion detected, which occurred in 28 of the 67 patients, and these were not scored.

#### 2.4.3. Statistical Evaluation

Multiple instances of two-sided paired *t*-tests [60] were performed throughout the analysis. Two data sets are assumed to be significantly different when p≤0.05 [61]. Linear regression was performed using a least-square fit [62] of a function of the form y=ax+b to the data characterized by the Pearson correlation coefficient r [63,64].

## 3. Results

### 3.1. Repetition and Voxel Rejection

**Figure 3:**
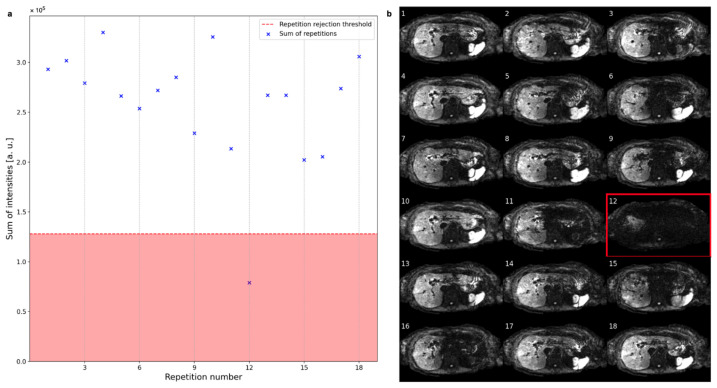
(**a**) The sum of intensities of one slice across all repetitions can vary substantially between repetitions. The red line marks the threshold under which slices get rejected. (**b**) The rejected slice at repetition number 12 suffers from severe global signal loss, while some other repetitions suffer from local signal loss.

Figure 3 shows a representative example of slice rejection. The sums of the intensities of a single slice for 18 repetitions are plotted in Figure 3a with the rejection region indicated by a red background. The slice at repetition 12 is rejected due to its overall low signal intensity, as is also apparent from Figure 3b.

**Figure 4:**
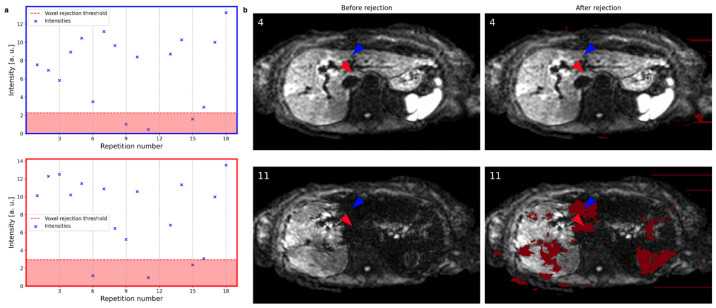
(**a**) The magnitude values of two voxels across all repetitions (excluding the previously rejected slice at repetition number 12) are shown for two exemplary voxels. The rejection threshold indicated by the red line and background depends on the spatial location. (**b**) Slices at repetitions 4 and 11 before and after the voxel rejection are shown with rejected voxels indicated in dark red. The colored arrowheads point to the voxels evaluated in (**a**) with their colors matching the color of the plots in (**a**). Repetition number 4 does not suffer from signal dropout, and consequently, no voxels inside the liver are rejected. Repetition number 11 suffers from signal loss, and patches of voxels are rejected.

Figure 4a shows the magnitude variation at two voxels across repetitions with their respective rejection thresholds indicated by the red line and background. The arrowheads indicate the corresponding voxel locations in Figure 4b. While both the red and the blue voxels exhibit high magnitude values in repetition number 4, their magnitude values in repetition number 11 (bottom figures) are severely reduced. Therefore, both voxels are rejected (dark red shade) in repetition number 11 and not in repetition number 4. Repetition number 4 does not suffer from signal dropout, and consequently, no voxels inside the liver are rejected. Repetition number 11 suffers from local signal loss effects, and patches of voxels are rejected, as indicated in dark red shade in Figure 4b. Some parts of the left liver lobe were spared from rejection due to localized signal loss in multiple repetitions. Though, the overall weights reflect the signal loss as presented in the next section.

### 3.2. Spatial Scaling of Average DWI

**Figure 5:**
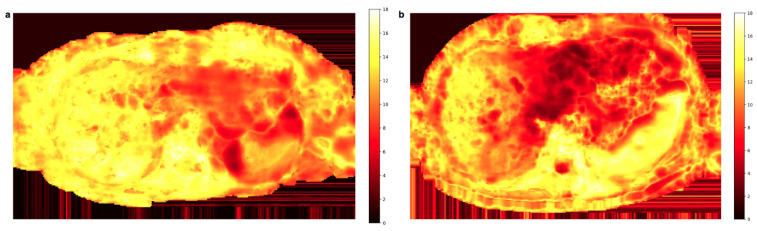
Two representative weight maps calculated with Equation (9) for the b600 diffusion-weighted images. Lower weight values reflect higher signal loss and mainly localize to the left liver lobe.

The weight map $W_{z}\left(x,y\right)$ computed for the same slice as in Figure 3 and Figure 4 is depicted in Figure 5a. The values in the region of the left liver lobe are smaller than those in the right liver, where the scaling factor mostly tends to the maximum value of 18 (the total number of averages). Another example of the weight map shown in Figure 5b also illustrates the concentration of lower weights to the motion-affected left liver.

**Figure 6:**
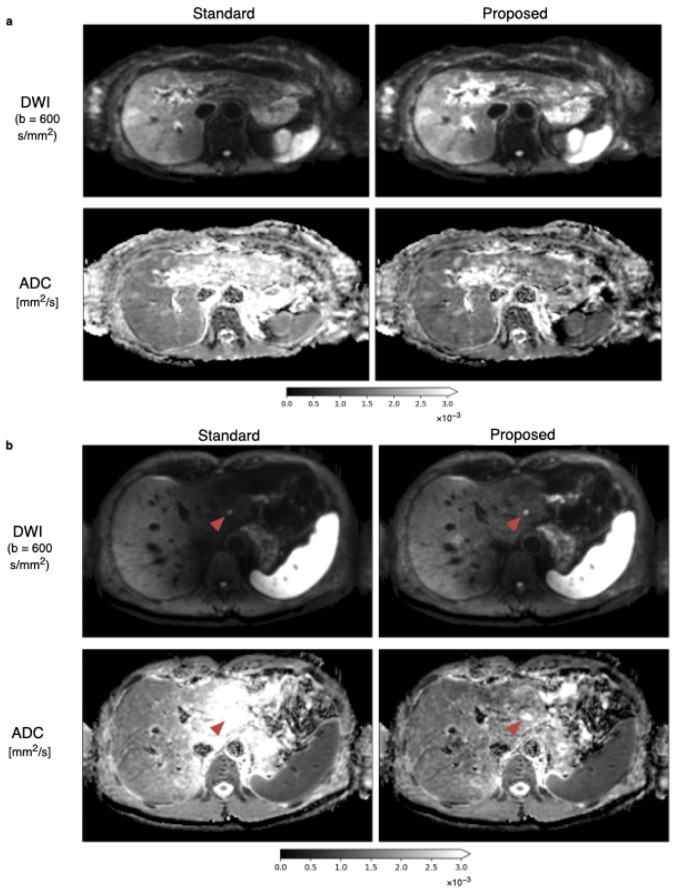
Average DWIs at b-values of $b=600\;{s/\mathrm{mm}}^{2}$ and corresponding ADC maps with the standard averaging and proposed methods. (**a**) An increase of intensity inside the left liver in the DW image in the top row for the proposed method compared to the standard averaging is observable, leading also to a more homogeneous appearance of the liver in the ADC images in the bottom row. (**b**) Another in vivo data set visualizes the ability of the proposed method to reduce signal loss in the left liver lobe in DWIs and to reduce the artificial overestimation of ADC. The lesion on the left side of the liver, which is marked with the red arrow, has improved visibility and is better defined in the DW image and particularly in the ADC image.

The diffusion-weighted images at the b-value of $b=600\;{s/\mathrm{mm}}^{2}$ and ADC maps from the standard and proposed methods with the weight map of Figure 5a applied are shown in Figure 6a. The left liver lobe appears brighter, and ADC overestimation is reduced. A second example case in Figure 6b illustrates the algorithm’s ability to mitigate the motion-induced signal loss in the left liver. With spatial scaling, the liver is more homogeneous in both the diffusion-weighted image and the ADC map. The reduction in signal loss appearance in the DW image gives better delineation of the lesion inside the left liver lobe.

### 3.3. Quantitative ADC Measurement

**Figure 7:**
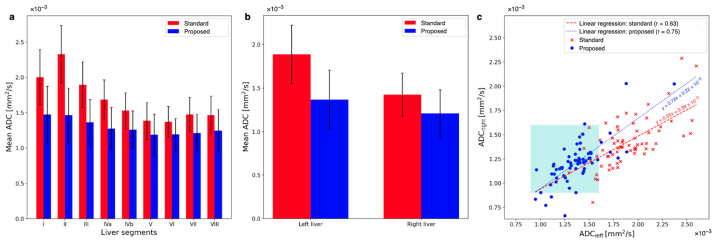
(**a**) A comparison of ADCs in the nine Couinaud liver segments reveals that ADCs in segments I, II, and III are overestimated the most, but they also they show the greatest correction to lower values after using the proposed method. ADCs of the segments of the right liver experience smaller changes toward lower values after using the proposed method, although they are significant. (**b**) The average ADC for all patients in the left liver is reduced by 38.2%, while in the right liver, the reduction is only 17.6%. This results in a more homogeneous liver appearance using the proposed method: with an ADC liver lobe ratio (ALR) of 1.13 ± 0.53 for the proposed method but 1.33 ± 0.47 for the standard method. (**c**) The comparison between left and right liver is plotted for all patients. The cohort of ADCs from the proposed method is notably more dense and shifted towards the lower left corner and inside the cyan square that indicates the range of ADCs of healthy liver parenchyma. Linear regression of both these cohorts yields a steeper slope that is closer to 1 for the ADCs using the proposed method.

Means and standard deviations of ADC values across patients are plotted for both averaging methods in Figure 7a. The standard averaging method yields higher ADC values in the left liver lobe than in the rest of the liver. The obtained ADC values within the left liver lobe are more stable across all liver segments when employing the proposed method. Two-sided paired *t*-tests show that the differences between the ADCs obtained from the two averaging methods are significant in all liver segments, with *p*-values listed in Table 1 next to the mean ADCs of both methods in the Couinaud liver segments.

When averaging the respective segments for the left (segments I–IVb) and the right (segments V–VIII) liver, an overestimation of the ADC in the left liver compared to the right liver can be observed when standard averaging is employed (see Figure 7b). Applying the proposed method reduces the overestimation of the ADC in the left liver. The mean ADC of the left liver is reduced by 38.2% by using the proposed method (p<0.01). With the proposed method, ADC in the right liver is also reduced, but only by 17.6% (p<0.001). The ratio of ADC of the left liver to ADC of the right liver (ADC liver lobe ratio, ALR) is $ALR_{\mathrm{standard}}=1.33\pm 0.47$ for the standard method and $ALR_{\mathrm{proposed}}=1.13\pm 0.53$ for the spatially scaled method. The ADCs in the left and the right liver are different with standard averaging (p=0.021) and the proposed method (p=0.020).

Figure 7c compares the ADCs of the left and right liver for all individual patients. Note that the majority of ADC values lie inside the expected range of $\left(0.9-1.6\right)\times 10^{-3}$ mm^2^/s only after applying the proposed method. This range is indicated in the plot by the cyan square. The slope of a linear regression between the left and right liver ADC values is closer to 1 for the proposed method: with 0.72 as compared to 0.55 with the standard method. Also, the Pearson-r correlation coefficient is greater for the ADC maps using the proposed method: with r=0.75 for the proposed and r=0.63 for the standard averaging method. The greater r-value indicates a higher linear correlation between the left and right liver lobes after applying the proposed algorithm. The ALRs of the individual patients with the proposed algorithm applied are significantly different from those of the standard averaged data set (p<0.001).

**Figure 8:**
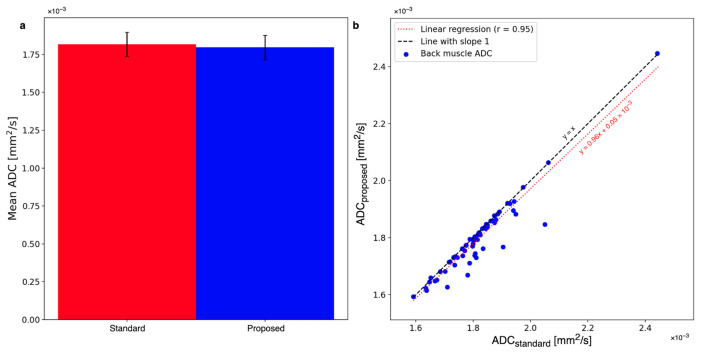
(**a**) The mean ADC in the back muscle changes from $1.82\pm 0.08\times 10^{-3}$ mm^2^/s to $1.80\pm 0.08\times 10^{-3}$ mm^2^/s. The change is statistically insignificant. (**b**) The shift towards smaller ADCs can also be observed when comparing the whole cohort of patients with standard averaging and the proposed method. The linear regression of the plot has a slope of 0.96.

The bias of the proposed method was tested by comparing ADC maps with the standard and proposed methods in the paraspinal muscle regions where motion is negligible. Figure 8a visualizes the mean ADCs averaged over both ROIs of the standard averaging and proposed methods. The mean ADCs are reduced on average from $1.82\pm 0.08\times 10^{-3}$ mm^2^/s to $1.80\pm 0.08\times 10^{-3}$ mm^2^/s. The ADC reduction is not statistically significant (p=0.31). Figure 8b plots the mean ADCs in the paraspinal muscles of all patients with the standard averaging and proposed methods. The linear regression with a slope close to 1 is rotated by a small amount from the y=x line, indicating a slight trend towards smaller ADCs.

### 3.4. Radiological Reading

The radiologist’s scores of the DWIs from standard averaging and the proposed method are compared in Table 2. The rows indicate the number of patient data sets in which the respective category scored higher, the same, or worse for the proposed spatially scaled averaging compared to standard averaging. There is statistically significantly higher liver homogeneity for the proposed method with a mean score of 3.13 ± 0.92 as compared to standard averaging with a mean score of 2.82 ± 0.89 (p=0.05). The improvement in the other categories is found to be not statistically significant for image quality (p=0.93), perceived SNR (p=0.51), and better lesion detection (p=0.53).

## 4. Discussion

The present work proposes a fast post-processing method to mitigate motion-induced signal loss in DWI of the liver, especially in the left lobe, relying on the acquisition of typically many repetitions. The two-step method deploys the rejection of slice and voxel outliers based on data-based thresholds before averaging the multi-repetition data deploying a scaled average. The proposed algorithm reduces the motion-induced signal loss in liver DWI and consequently also corrects for ADC overestimation.

Outlier rejection had been proposed earlier [40] using a different method. In the proposed method, the rejection step discards noise-level data with simple thresholding steps on the repetition and voxel levels. Rejected data are excluded from further processing to improve the noise performance of the method. Both the slice and voxel rejection procedures employ tunable parameters controlling the amount of data to be rejected. Optimal rejection parameters can be found by evaluating the trade-off between the reduction in motion-induced signal loss and the SNR of the averaged image. The trade-off stayed stable when the thresholds of the rejections fell below the median of the data. Thus, the rejection parameters in the current study were fixed to empirical values. In another study, a voxel-wise exclusion algorithm was optimized regarding an exclusion threshold [42].

The spatial scaling factor $w_{z,i}\left(x,y\right)$ in Equation (9) reflects the motion-induced signal loss at each spatial location in the DWIs. Estimating these spatial scaling factors requires reference DWIs that are minimally affected by motion-induced signal loss. In this work, such reference images were chosen as the MIP across all acquired repetitions. Another choice of reference images such as the sum of squares (or higher power) of all repetitions or the average of manually selected good images can also be used [41,43]. The choice of reference images dictates the trade-off between SNR performance, processing time and manual work, and the residual motion-induced signal loss in the final averaged images. More specifically, the choice of a noisy MIP as reference leads to scaling factors that are affected by noise but reflect the signal loss well. The sum-of-squares images have high SNR but still retain significant signal loss, especially in the case where most of the repetitions are corrupted. While manual selection of good images arguably gives the best possible reference, the selection must be carried out for each and every data set separately and is time-consuming and labor intensive. With the choice of an MIP as the reference, the proposed algorithm dampens the effect of noise by operating on low-pass-filtered images and setting all scaling factors larger than 0.8 to 1.

Current post-processing approaches to mitigate motion-induced signal loss in DWI rely on heuristic methods that penalize the contributions of signals with lower magnitudes to the final combined images. In contrast, the proposed method is based on a mathematical formulation of the effect of motion-induced signal loss on each repetition (Equation (2)), allowing for more flexible ways to combine the repetitions. Future work can find different ways to combine individual repetitions based on the proposed model.

Quantitatively, the proposed algorithm significantly reduced the ADC bias towards elevated values in the left liver lobe, shifting it towards the reported range of healthy liver ADC ($0.9-1.6\times 10^{-3}$ mm^2^/s) [34,65,66]. Other studies also showed that reducing the bias in the liver with post-processing methods is possible [29,41,44]. Moreover, for the whole cohort of patient data, a trend towards a more homogeneous liver was observed, as reflected by a slope closer to 1 for the regression comparing left and right liver ADCs. The significant change in ADC in the right liver lobe can be attributed to motion affecting this area, albeit to a lesser extent than the left liver lobe [23]. Thus, the proposed method reduces the signal-loss artifact due to all kinds of motion and not only the cardiac-motion-induced signal loss in the left liver lobe. Additionally, the proposed method never decreases the signal compared to the standard averaging method and is likely to increase it, especially for higher b-values, leading to a corresponding reduction in ADC. However, in the paraspinal muscle, where motion is minimal, the ADC of the proposed method is not significantly different from the ADC of the standard averaging method.

The proposed method was evaluated qualitatively by a radiologist, showing that the proposed method produces more homogeneous liver DWIs compared to the standard averaging method. Improving the homogeneity of liver DWIs is essential for diagnostic accuracy, and this was achieved in 35.8% of all evaluated patient cases using the proposed algorithm. The homogeneous appearance of healthy liver parenchyma is essential for diagnostic accuracy. In cases of motion-induced signal loss, the left liver can be excluded from diagnosis due to the low image quality [67]. The proposed method can effectively mitigate this issue and provide images with improved homogeneity of healthy liver parenchyma. In some patient cases, the proposed method also improved lesion detection, demonstrating its potential value in clinical routine. However, since only 28 out of 67 patients in the present cohort had lesions detected in the liver, and some of the lesions were only present in parts of the liver where the signal loss was less severe, further larger-sized studies with a dedicated cohort of patients with diagnosed hepatic lesions are needed to assess the potential enhancement in lesion detection. While another study was conducted on a similarly sized cohort of patients with hepatic lesions [42], there is no other study to the best of our knowledge that used clinical reading as evaluation of post-processing methods for the reduction in motion-induced signal loss in liver DWI on a similar patient cohort size.

Several additional processing and analysis techniques can further improve the proposed method. In diffusion imaging, motion and eddy currents can cause misregistration across repetitions. Also, there are multiple possible sources for other systematic errors that can be addressed next to the motion-induced signal loss, such as the gradient non-linearity or the spatial distribution of b-values. [18,19,20,21,68,69]. Though, the effective ADC bias due to motion observed in this study is substantially greater than the effects of any higher-order sources of bias. Therefore, the proposed method focuses on reducing motion-induced signal loss with the possibility to include higher-order effects in a future study. Including higher-order effects in the correction algorithm can be a direction of future work.

Since motion-induced signal loss is expectedly patch-wise and of low resolution, the rejection of data and the estimation of the scaling factors were performed on low-pass-filtered data. Therefore, the effect of misregistration on the estimated scaling factors is reduced. Nevertheless, registration before further processing might further improve the precision of the scaling factors and reduce blurring in the final averaged image. Furthermore, the proposed method assumes that the only difference across repetitions of each b-value is the motion-induced signal loss. While this assumption is true most of the time, the existence of other types of artifacts that change from repetition to repetition at each b-value would compromise the performance of the method. These artifacts, if any, must be addressed before applying the spatial scaling method. Penultimately, the proposed approach is a data-driven approach and thus does not guarantee the complete removal of motion-induced ADC quantification bias. Finally, the proposed approach would not work in cases where severe motion artifacts appear across all repetitions. In such cases, the use of MCDE waveforms would be preferred.

A complete understanding of the proposed algorithm might benefit from analyzing the SNR performance. While the slice and voxel rejection steps can help with noise reduction, the spatial scaling step might increase noise. The noise performance of the spatial scaling step depends on the choice of reference images. A Monte Carlo simulation can give insights into intermediate and final SNR behavior of the method. The behavior of the rejection steps might change in cases of free-breathing acquisitions because more outliers are expected compared to respiratory-triggered scans. Therefore, the effect of SNR benefit might be greater under free-breathing conditions. However, the robustness has to be evaluated first since too many repetitions containing outliers are likely to interfere with the rejection process.

The study has some limitations that should be noted. First, the significant change in ADC in the right liver lobe after applying the proposed method needs to be fully explained. Pulsation artifacts from vessels could partly explain this observed reduction in ADC. Second, the thresholds for outlier rejection were chosen empirically, which may limit reproducibility and the possibility of applying the method to other datasets, particularly since only data from a single scanner were used. A more thorough investigation of the influence of those parameters and of a more robust way to determine them can be a direction for future work. Adaptive thresholding [40] or optimization of a threshold [42] might be beneficial. Third, ADC values were only compared to the reported range of healthy liver parenchyma ADCs in the literature, and there was no ground truth ADC for comparison to assess the outcome of the proposed method. Last, the clinical reading was performed by only a single radiologist, rendering it impossible to evaluate the quality of scoring or to perform an inter-rater comparison.

## 5. Conclusions

A spatially scaled averaging approach with prior outlier rejection for multi-repetition liver DWI was developed to mitigate the motion-induced signal loss in repetition-combined DW images. Quantitative and qualitative assessments show the superior performance of the proposed method in achieving more homogeneous liver DWI and reduction of ADC overestimation, especially in the left liver lobe, as compared to the standard averaging method. This can potentially improve clinical diagnostics, as the left liver lobe can be assessed more precisely. The algorithm is easy to implement and has a fast processing time. The proposed model of the effect of motion-induced signal loss on each repetition and on the final combined image can be used as the base for other advanced repetition combination approaches that minimize signal loss.

## Figures and Tables

**Table 1:** The mean ADCs over all subjects are reduced in every Couinaud liver segment. The *p*-values of differences in ADCs between standard averaging and the proposed methods in the different liver segments all show statistical significance.

Liver Segment	ADC_standard_ [mm^2^/s]	ADC_proposed_ [mm^2^/s]	*p*-Value
I	1.97 ± 0.35	1.71 ± 0.36	9.0 × 10${}^{-20}$
II	2.25 ± 0.33	1.82 ± 0.33	2.5 × 10${}^{-19}$
III	1.87 ± 0.27	1.60 ± 0.28	1.3 × 10${}^{-12}$
IVa	1.70 ± 0.25	1.48 ± 0.25	1.7 × 10${}^{-13}$
IVb	1.58 ± 0.22	1.43 ± 0.23	1.2 × 10${}^{-8}$
V	1.43 ± 0.23	1.32 ± 0.24	5.9 × 10${}^{-9}$
VI	1.42 ± 0.18	1.31 ± 0.18	2.4 × 10${}^{-5}$
VII	1.54 ± 0.20	1.40 ± 0.21	3.8 × 10${}^{-4}$
VIII	1.51 ± 0.23	1.38 ± 0.24	8.4 × 10${}^{-6}$

**Table 2:** Radiological reading scores for all patient data sets for the standard and proposed methods in four categories. Liver homogeneity scored significantly better, showing an improvement in 24 out of 67 cases (p=0.05). Improvements in the other three categories were not statistically significant.

	Image Quality	Liver Homogeneity	Perceived SNR	Lesion Detection
**Better**	1	24	13	3
**Same**	66	39	48	25
**Worse**	0	4	6	0

## Data Availability

Data are available upon request and pending institutional review.

## Abbreviations

The following abbreviations are used in this manuscript:

ADC	Apparent Diffusion Coefficient
ALR	Apparent diffusion coefficient liver Lobe Ratio
DW	Diffusion-Weighted
DWI	Diffusion-Weighted (magnetic resonance) Imaging
EPI	Echo-Planar Imaging
FOV	Field Of View
MCDE	Motion-Compensated Diffusion Encoding
MRI	Magnetic Resonance Imaging
ROI	Region Of Interest
SNR	Signal-to-Noise Ratio
TE	Echo Time
T2	T2 relaxation time
